# Editorial: AI in single-cell biology

**DOI:** 10.3389/fbinf.2026.1873145

**Published:** 2026-05-19

**Authors:** Martin Hemberg

**Affiliations:** Brigham and Women’s Hospital, Harvard Medical School, Boston, MA, United States

**Keywords:** AI, editorial, large langauge models, single cell, spatial transcriptomics

Single-cell and spatial technologies are now established in virtually every area of biomedical research, and they are a powerful tool for characterizing cellular heterogeneity. However, the dimensionality, noise and complexity of single cell data makes it very challenging to work with. Given the recent advances in AI and its demonstrated ability to process data in domains such as text, images, and protein structures, it is only natural that scientists have sought to apply these methods to single cell data. The “*AI in Single-Cell Biology*” Research Topic collects a set of timely contributions that together survey the state of the art, introduce new algorithmic tools, and demonstrate practical pipelines for integrating single-cell and spatial omics. The five papers in the Research Topic—a broad methods review, a spatial–transcriptomics primer, a spatial-domain identification model built on large language models, a multi-omics vaccine-response study, and a practical metrics paper for CITE-seq quality control—illustrate some of the many opportunities that AI provides for computational analysis of single cell data.

The framing review, “*Applications of AI to single-cell and spatial transcriptomics: current state-of-the-art and challenges*” by Tchatchoua Ngassam et al. provides a comprehensive overview of where AI has delivered and where it has yet to (Tchatchoua Ngassam et al.). The authors evaluate ten central tasks—dimensionality reduction, cross-dataset integration, denoising, augmentation, deconvolution, cell-cell interaction inference, RNA velocity, ATAC–RNA integration, modality integration, and ST alignment—and contrast deep learning solutions against classical statistical or heuristic alternatives. The conclusion is that deep learning models offer compelling power for large, well-curated datasets and specialized tasks, but many generative approaches remain insufficiently benchmarked for discovery work. Importantly, the review flags two recurring practical limitations: (1) data scarcity and domain shift across platforms, which degrade generalization, and (2) limited interpretability of end-to-end deep pipelines. The latter in particular is a challenge for AI models across all domains, and it once again highlights the need for the development of interpretable models.

Complementing that high-level perspective, “*Machine learning approaches for biomarker discovery using single-cell RNA sequencing*” by Dewa et al. provides a broad overview of current machine learning workflows for biomarker discovery using scRNA-seq data. The article places particular emphasis on feature selection methods, supervised learning strategies, classification metrics, and downstream biological validation. Rather than focusing solely on predictive performance, the review highlights the importance of interpretability, biological relevance, and methodological diversity across cell-level and patient-level approaches. By outlining common analytical frameworks and evaluation strategies, the paper offers practical guidance for researchers interested in translating single-cell analyses into biologically meaningful and clinically relevant biomarkers.

In recent years spatial transcriptomics technologies have made substantial strides, as several commercial platforms have become available. The data provided holds the potential for many more novel insights compared to dissociated single cell data. However, current computational models are not able to take full advantage of this data, and applying AI models to spatial transcriptomics is an active area of research. The original research paper “*SpaLLM: a general framework for spatial domain identification with large language models*” recasts spatial domain discovery as a sequence modeling problem solvable with transformer-style LLM components (Zou et al.). SpaLLM abstracts local spatial neighborhoods and their transcriptomic signatures into tokenized representations that are fed to a pretrained language model architecture, enabling contextualized embeddings that capture both histological neighborhood structure and gene expression patterns. The method demonstrates robust domain segmentation across diverse tissue types and offers interpretability through attention maps that highlight features driving domain boundaries. SpaLLM exemplifies the growing trend of repurposing successful architectures from natural language processing and vision to handle structured biological inputs. A key aspect, as alluded to above, is the emphasis on interpretability through attention maps and neighborhood statistics.

Another important trend in the field has been the development of multi-modal assays that can profile individual cells in more than one way. “*Comprehensive analysis of multi-omics vaccine response data using MOFA and Stabl algorithms*” presents a case study applying matrix factorization (MOFA) and sparse stabilization (Stabl) methods to paired single-cell and protein readouts from vaccine cohorts (Gupta et al.). The paper demonstrates how latent factor models, combined with sparsity-promoting selection, can reveal coordinated immune programs predictive of response. The work highlights practical pipeline elements—careful batch correction, selection of biologically motivated priors, and downstream validation on independent cohorts—illustrating how AI tools can be integrated into rigorous experimental design to yield replicable biological hypotheses.

Finally, the methods paper “*Quantitative measures to assess the quality of cellular indexing of transcriptomes and epitopes by sequencing data*” tackles a lower-level but essential problem: quality assessment for combined transcriptomic and epitope (CITE-seq) measurements. Sun et al. propose a suite of quantitative metrics that capture indexing efficiency, ambient background, doublet contamination, and epitope assignment fidelity. These metrics are accompanied by practical thresholds and visualization diagnostics, making them immediately usable in preprocessing pipelines. Quality control is a key component of any bioinformatics pipeline, and it is especially important when preparing data to be used by AI models. As such, careful analyses of the pre-processing is essential for the success of the field.

Taken together, these five contributions highlight several convergent themes. First, hybrid pipelines that combine domain-aware feature engineering and classical statistical regularization with modern ML architectures are favored over purely end-to-end black-box methods. Such a strategy is required to enhance interpretability and without interpretable models it will be much more challenging to discover new biology. Second, spatial transcriptomics continues to be a major frontier, but the field has still not determined what are the best representations and models for this data. Repurposing of existing models, e.g., tokenization for LLMs, show promise, but data limitations and modality mismatch remain impediments. Third, rigorous benchmarking and quality control are non-negotiable: papers that pair algorithmic innovation with clear validation strategies and QC standards are immediately more valuable to practitioners.

The Research Topic also implicitly sets an agenda. The field needs larger, standardized, multimodal datasets with matched histology and spatial context, stronger community benchmarks tied to biological endpoints. Mere quantification of reconstruction loss is insufficient, and biological aspects and interpretation must be considered as well. For practitioners, the practical takeaway is to prioritize transparent, well-validated hybrid workflows: start with robust QC, use interpretable feature extraction and latent factor models where possible, and employ deep architectures judiciously for tasks only when sufficient data and clear validation targets are available.

In sum, the “*AI in Single-Cell Biology*” Research Topic marks a maturing field: AI is no longer a curiosity at the edge of single-cell research but a practical toolset whose adoption must be governed by statistical rigor, interpretability, and careful experimental design. These five papers offer both a roadmap and a toolbox for researchers aiming to turn single-cell data into reliable biological insight.

